# Scleral Buckling for Primary Retinal Detachment: Outcomes of Scleral Tunnels versus Scleral Sutures

**DOI:** 10.18502/jovr.v16i3.9434

**Published:** 2021-07-29

**Authors:** Matthew R. Starr, Edwin H. Ryan, Anthony Obeid, Claire Ryan, Xinxiao Gao, Malika L. Madhava, Sean M. Maloney, Adam Z. Adika, Krishi V. Peddada, Kareem Sioufi, Luv G. Patel, Michael J. Ammar, Nora J. Forbes, Antonio Capone Jr., Geoffrey G. Emerson, Daniel P. Joseph, Dean Eliott, Carl D. Regillo, Jason Hsu, Omesh P. Gupta, Yoshihiro Yonekawa, for the Primary Retinal Detachment Outcomes (PRO) Study Group

**Affiliations:** ^1^Wills Eye Hospital, Mid Atlantic Retina, Thomas Jefferson University, Philadelphia, PA, USA; ^2^VitreoRetinal Surgery, Minneapolis, MN, USA; ^3^Lewis Katz School of Medicine at Temple University, Philadelphia, PA, USA; ^4^Department of Ophthalmology, Drexel University College of Medicine, Philadelphia, USA; ^5^Associated Retinal Consultants, Oakland University William Beaumont School of Medicine, Royal Oak, MI, USA; ^6^The Retina Center, Minneapolis, MN, USA; ^7^The Retina Institute, St. Louis, MO, USA; ^8^Massachusetts Eye and Ear, Harvard Medical School, Boston, MA, USA

**Keywords:** Rhegmatogenous Retinal Detachment, Scleral Buckle, Scleral Suture, Scleral Tunnels, Strabismus

## Abstract

**Purpose:**

There are primarily two techniques for affixing the scleral buckle (SB) to the sclera in the repair of rhegmatogenous retinal detachment (RRD): scleral tunnels or scleral sutures.

**Methods:**

This retrospective study examined all patients with primary RRD who were treated with primary SB or SB combined with vitrectomy from January 1, 2015 through December 31, 2015 across six sites. Two cohorts were examined: SB affixed using scleral sutures versus scleral tunnels. Pre- and postoperative variables were evaluated including visual acuity, anatomic success, and postoperative strabismus

**Results:**

The mean preoperative logMAR VA for the belt loop cohort was 1.05 ± 1.06 (Snellen 20/224) and for the scleral suture cohort was 1.03 ± 1.04 (Snellen 20/214, *p* = 0.846). The respective mean postoperative logMAR VAs were 0.45 ± 0.55 (Snellen 20/56) and 0.46 ± 0.59 (Snellen 20/58, *p* = 0.574). The single surgery success rate for the tunnel cohort was 87.3% versus 88.6% for the suture cohort (*p = *0.601). Three patients (1.0%) in the scleral tunnel cohort developed postoperative strabismus, but only one patient (0.1%) in the suture cohort (*p = *0.04, multivariate *p = *0.76). All cases of strabismus occurred in eyes that underwent SB combined with PPV (*p = *0.02). There were no differences in vision, anatomic success, or strabismus between scleral tunnels versus scleral sutures in eyes that underwent primary SB.

**Conclusion:**

Scleral tunnels and scleral sutures had similar postoperative outcomes. Combined PPV/SB in eyes with scleral tunnels might be a risk for strabismus post retinal detachment surgery.

##  INTRODUCTION

The use of scleral buckles (SBs) to repair rhegmatogenous retinal detachments (RRDs) was pioneered by Custodis in 1949, with the first reported scleral buckling procedure performed in the United States in 1951 by Schepens.^[1]^ Schepens' initial technique describes a lamellar dissection of the sclera and placement of an element with external diathermy for retinopexy.^[1,2]^ An initial report had a success rate of 65%,^[2]^ but over the years, scleral buckling has evolved and lamellar dissection is rarely performed. Currently, the most commonly performed is the use of scleral suturing to secure the SB directly on the surface of the sclera, but the use of scleral tunnels to affix the encircling buckle to the sclera is a popular technique as well.^[3]^ The selection largely depends on surgeon's preference, and little data is available regarding comparative efficacy and outcomes.^[3]^

Diplopia from strabismus following SB surgery is often temporary, but chronic or permanent strabismus may also occur and is a well-known complication, with a reported incidence between 5% and 25%.^[4]^ The management of postoperative strabismus usually begins with prism therapy which may resolve the strabismus in the majority of patients, while other patients may require strabismus surgery or buckle removal.^[4,5]^ Similar to the lack of reports examining anatomic outcomes following the use of scleral tunnels or scleral sutures, there have been no reports assessing the development of strabismus comparing these two techniques for buckle fixation.

The purpose of this paper is to present the anatomic outcomes following scleral buckling surgery comparing scleral tunnels to scleral fixated sutures, but additionally, to assess the development of postoperative strabismus between these two modalities.

##  METHODS

The Primary Retinal Detachment Outcome (PRO) study is a multicenter, interventional, retrospective cohort study of patients who underwent repair of noncomplex primary RRD from January 1, 2015 through December 31, 2015 from VitreoRetinal Surgery in Minneapolis, The Retina Center in Minneapolis, The Retina Institute in St. Louis, Associated Retinal Consultants/William Beaumont Hospital in Detroit, Mass. Eye & Ear in Boston, and Mid Atlantic Retina/Wills Eye Hospital in Philadelphia.^[6]^ Institutional review board approval was obtained at each participating institution, and the study complied with the Health Insurance Portability and Accountability Act of 1996 and adhered to the tenets of the Declaration of Helsinki.

This report is a subgroup analysis of the PRO study. We examined the outcomes of patients who received SBs (either primary scleral buckling or in combination with vitrectomy), and compared visual and anatomic outcomes, as well as the rates of postoperative strabismus, as defined as ocular misalignment. Complex retinal detachments including retinal detachments that had previously undergone repair, tractional retinal detachments, and retinal detachments due to inflammation or endophthalmitis were excluded. Eyes with fewer than three months postoperative follow-up were excluded, as were eyes where the scleral suture or scleral tunnel metric was not recorded. Additionally, eyes that underwent vitrectomy without SB, non-encircling SB surgery, pneumatic retinopexy, or laser barricade were excluded. Eyes that underwent scleral buckling procedures after the initial procedures, namely reoperations for recurrent retinal detachment, were excluded as well.

Detailed demographic, preoperative, intraoperative, and postoperative follow-up variables were collected from each site using the secure online REDCap database. The primary outcomes considered were single surgery anatomic success, postoperative visual acuity, and the development of postoperative strabismus that was noted at the final postoperative visit, which must have taken place more than three months following the surgery. Single surgery anatomic success was defined as posterior retinal attachment with no tamponade present, and no presence of subretinal fluid which could spread at three months postoperatively. Stable, localized subretinal fluid following primary SB was not considered a failure. Sub-analyses included outcomes of SB band type and eyes with only primary SB surgery without pars plana vitrectomy (PPV).

For statistical analysis, we used JMP software version 15.0 (SAS Institute, Cary, NC). For within-group comparisons between baseline and final metrics, a paired *t*-test was used, and for comparisons between groups, the Wilcoxon rank-sum test was performed. Group comparisons of the categorical data were performed using the Fisher's exact test. A repeated-measures mixed model regression analysis was performed for multivariate analysis comparing SB sutures versus scleral tunnels controlling for surgeon, type of surgery (PPV with SB versus primary SB), buckle type, postoperative epiretinal membrane, and cataract status was performed. A *p-*value < 0.05 was considered to be statistically significant.

##  RESULTS

There were 1,148 eyes that met the inclusion criteria and underwent SB or combined PPV and SB for primary, noncomplex RRD, with 289 eyes (25.2%) undergoing SB and 859 eyes (74.8%) undergoing combined PPV with SB. The mean age of the patients was 56.4 ± 14.1 years with 38.4% being female. The mean follow-up after surgery was 365 ± 186 days. The mean preoperative logMAR VA for all eyes was 1.04 ± 1.05 (Snellen VA 20/219) and the mean postoperative logMAR VA was 0.46 ± 0.58 (Snellen 20/58, *p*-value < 0.0001). The single surgery success rate for all eyes was 88.2%.

Of these 1,148 eyes, 302 had the encircling SB affixed to the sclera with scleral tunnels, while 846 eyes had scleral sutures placed. Demographic data for these cohorts are detailed in Table 1. Briefly, the mean preoperative logMAR VA for the belt loop cohort was 1.05 ± 1.06 (Snellen 20/224) and for the scleral suture cohort was 1.03 ± 1.04 (Snellen 20/214, *p* = 0.846). The mean postoperative logMAR VA for the belt loop cohort was 0.45 ± 0.55 (Snellen 20/56) and for the scleral suture cohort was 0.46 ± 0.59 (Snellen 20/58, *p* = 0.574).

**Table 1:** Demographics and visual and anatomic outcomes comparing scleral tunnels versus scleral sutures in fixation of scleral buckle during retinal detachment surgery for both scleral buckle surgery and pars plana vitrectomy combined with scleral buckle


	**Scleral tunnels (** ***n*** ** = 302)**	**Suture (** ***n*** ** = 846)**	***P*** **-value**
**Age**	57.7 ± 13.6	55.9 ± 14.3	0.04 ${}^{*}$
**Sex (Female)**	117 (38.8%)	314 (37.1%)	0.63
**Preoperative logMAR (Snellen)**	1.05 ± 1.06 (20/224)	1.03 ± 1.04 (20/214)	0.85
**Postoperative logMAR (Snellen)**	0.45 ± 0.55 (20/56)	0.46 ± 0.59 (20/58)	0.57
***P*** **-value**	< 0.0001 ${}^{*}$	< 0.0001 ${}^{*}$	
**Single surgery success rate**	87.3%	88.6%	0.60
**Postoperative strabismus **	3 (1.0%)	1 (0.1%)	0.02 ${}^{*}$
**Eyes with encircling band only**	278 (92.1%)	816 (96.5%)	0.01 ${}^{*}{}^{+}$
**Follow-up (days)**	368 ± 185	363 ± 187%	0.99
logMAR, logarithm of the minimum angle of resolution ${}^{*}$ Statistically significant value ${}^{+}$ Fischer's Exact test			

**Table 2:** Analysis of buckle type between the belt loop and scleral suture cohort


**Buckle element**	**Scleral tunnels (** ***n*** ** = 302)**	**Suture (** ***n*** ** = 846)**
**240 **	53 (17.6%)	57 (6.7%)
**40 **	2 (0.7%)	36 (4.3%)
**41 **	246 (81.4%)	524( 62.0%)
**42 **	1 (0.3%)	183 (21.6%)
**Other**	0 (0%)	46 (5.4%)

**Table 3:** Demographics and visual and anatomic outcomes comparing scleral tunnels versus scleral sutures in fixation of a scleral buckle during retinal detachment surgery in only eyes undergoing scleral buckling only


	**Scleral tunnels (** ***n*** ** = 61)**	**Suture (** ***n*** ** = 226)**	***P*** **-value**
**Mean age (yr)**	44.7 ± 16.8	47.1 ± 16.1	0.21
**Sex (Female)**	20 (32.8%)	112(49.6%)	0.02 ${}^{*}$
**Preoperative logMAR (Snellen)**	0.34 ± 0.57 (20/44)	0.55 ± 0.74 (20/71)	0.003 ${}^{*}$
**Postoperative logMAR (Snellen)**	0.27 ± 0.41 (20/37)	0.27 ± 0.37 (20/37)	0.812
**Single surgery success rate**	85.2%	89.8%	0.32
**Postoperative strabismus rate**	0 (0%)	0 (0%)	1.00
**Follow-up (days)**	373 ± 210	352 ± 173	0.58
logMAR, logarithm of the minimum angle of resolution ${}^{*}$ Statistically significant value			

The single surgery success rate for the belt loop cohort was 87.3% versus 88.6% for the suture cohort (*p = *0.601). Three eyes (1.0%) in the belt loop cohort developed postoperative strabismus while only one eye (0.1%) developed postoperative strabismus in the suture cohort (*p = *0.04, Table 1). On logistic regression analysis accounting for surgeon identification (univariate analysis, *p = *0.38), type of surgery (PPV with SB versus SB, *p = *0.12), preoperative macular detachment status (*p = *0.49), postoperative epiretinal membrane formation (*p = *0.33), and postoperative cataract formation (*p = *0.51), there was no longer any significant difference in the development of postoperative strabismus with a *p-*value of 0.76. When accounting for the type of anesthesia (retrobulbar, sub-tenon, or general anesthesia), there was also no difference in the postoperative strabismus (*p *= 0.17). The distribution of buckle types is presented in Table 2, with the most common buckle used in each cohort being a 41 band. As one may expect, the belt loop cohort received smaller bands (more 240 and 41) compared with sutured bands (42) (*p* < 0.0001).

When analyzing only those eyes that had SBs placed (without vitrectomy), there were 287 cases. Of these, there were 61 (21.3%) with scleral tunnels and 226 (78.3%) with scleral sutures. There was no difference in single surgery success rate, postoperative visual acuity, or postoperative strabismus in this cohort (Table 3). There were no cases of strabismus following scleral buckling without vitrectomy (*p* = 0.02).

During the follow-up period, there were three cases of SB removal, one for infection and two for symptomatic diplopia. The buckles removed for symptomatic diplopia belonged to both the belt loop and scleral suture cohorts. The belt loop patient had the buckle removed seven months following the surgery while the scleral suture patient had it removed five months following the surgery. Both patients received a 41 band and underwent combination PPV + SB. The patient with the infected buckle received a 4050 band, was also in the PPV + SB cohort, had the buckle affixed with scleral sutures, and the patient had the buckle removed eight months following the surgery. There were no cases of buckle extrusion or intraoperative perforation in either cohort reported.

##  DISCUSSION

The routine method of SB fixation is typically dependent on the surgeon's preference. Scleral sutures can be used to anchor the buckle element to the scleral surface, either to secure an encircling band in place or to imbricate a segmental element into the eye wall. The vast majority of eyes received encircling bands in this study. Encircling bands can also be secured to the scleral via scleral tunnels, which are fashioned by creating partial thickness scleral tunnels in all four quadrants. The band is then passed through the scleral tunnels. Scleromalacia may bias a surgeon toward scleral sutures, but for routine placement, surgeon preference likely plays the biggest role in the decision to secure the buckle via sutures or scleral tunnels. There is very limited comparative data on the two techniques, and the present study represents the first large-scale study to examine the question.

A recent small study of 35 eyes examined the outcomes following combined PPV with SB surgery and compared scleral tunnels versus scleral sutures and found no difference in anatomic outcomes with no cases of buckle extrusion or infection.^[7]^ Similarly, the visual and anatomic outcomes were comparable in our significantly larger cohort. The single surgery success rate for all eyes in our series was 88.2% and when analyzed by fixation of the encircling band, there was no difference in the anatomic outcomes or visual acuity.

The use of scleral tunnels to secure an SB, though, appeared to be associated with a higher rate of postoperative strabismus in our study. However, when accounting for other metrics that may account for postoperative strabismus on multivariate analysis such as surgical approach or buckle type, there was no difference in postoperative strabismus. Interestingly, all cases of strabismus occurred in eyes that underwent combined scleral buckling and vitrectomy. This combination approach might have led to increased orbital inflammation and scarring of the periorbital tissue leading to strabismus. There were no cases of strabismus in eyes that received a primary buckle. Perhaps this cohort is better suited to identify any differences in outcomes in vision, anatomic success, or strabismus by excluding PPV. There were no differences in any metrics between scleral tunnels and scleral sutures. Certainly, this finding may support the conclusion that combination surgery is the inciting factor for strabismus.

There was a difference in the buckle element chosen between the two cohorts. Sutures theoretically have more flexibility in the size of buckle that can be placed with ease. For example, larger elements would require large scleral tunnels, which may be technically challenging and at higher risk for flap amputation or dissections going too deep. It would be technically easier to place sutures to accommodate larger elements. This trend was seen in our study as well, where the 42 band (4 mm wide, 1.25 mm thick) was sutured more frequently, while the 240 (2.5 mm wide, 0.6 mm thick) and 41 (3.5 mm wide, 0.75 mm thick) bands were used for scleral tunnels more often. It would be more plausible that the wider buckle, the 42 band, would be associated with a higher incidence of postoperative strabismus, but these were hardly used in the belt loop cohort and much more common in the scleral suture cohort (See Table 2), which had lower risk of postoperative strabismus. The mechanism of this counter intuitive finding is difficult to explain.

In our series, both scleral tunnels and scleral sutures were successful in repairing retinal detachment. In the previous smaller study of 35 eyes by Landa and colleagues, they also reported no buckle complications and additionally there were no cases of postoperative strabismus in either of their cohorts.^[3]^ Previous studies have found an incidence of postoperative strabismus following retinal detachment surgery to range from 5% to 25%.^[4,8,9]^ This is much higher than seen in our series, in which < 1% of patients developed postoperative strabismus. Perhaps, our study did not capture patients with minor strabismic deviations and only captured those patients with symptomatic strabismus with significant prismatic deviations.

There are several hypotheses regarding the etiology of postoperative strabismus following retinal detachment surgery, the majority of which are felt to be mechanical.^[4,10]^ Direct interference of the muscle due to the SB is certainly plausible (and of course if the rectus muscle is split), but some have also proposed direct muscle injury or disinsertion of the muscle as well as scarring of the orbit may also lead to postoperative strabismus.^[4,5][11][12][13]^ Conceivably, SB positioning may play a minor role compared to postoperative orbital inflammation and scarring as no patients in the primary SB cohort developed strabismus and it was only seen in those eyes that had combined PPV and SB. Previous studies, though, have shown a similar incidence between SB and PPV, with SB patients having slightly larger prismatic deviations.^[14]^ Additionally, the type of anesthesia may play a role in postoperative strabismus with direct inoculation of the rectus muscle with anesthesia, however, we did not see an effect on postoperative strabismus in our series between the types of anesthesia (retrobulbar versus sub-tenon's versus general anesthesia, *p = *0.15).

This study is limited inherently by its design as a retrospective analysis. This study was also a sub-analysis of a larger dataset not specifically designed to address the development of strabismus and perhaps lead to such low reported rates of strabismus. Additionally, as with any surgical outcomes study with several surgeons, a number of intraoperative factors cannot be accounted for that certainly could bias the results, such as suture or scleral tunnel length and depth, and how the buckle is manipulated around the extraocular muscles, including the amount of Tenon's capsule dissection. However, we controlled for surgeon ID, which theoretically would account for differences in surgical techniques. Another consideration when evaluating the data is that the vast majority of eyes in this cohort received encircling bands, as opposed to segmental elements. The dimensions of commonly used segmental buckles are relatively larger (usually tires or sponges), though they are usually not placed 360º around the eye if used in isolation. Therefore, the data in the present study may or may not be generalizable to segmental buckles that are imbricated in limited areas of the sclera. That being said, segmental elements are most often sutured in place, because imbrication is required to optimize the buckling effect, rather than encircling bands that provide elevation by tightening the band circumference. It is also certainly possible that combination PPV/SB is the inciting factor, rather than the method of affixing the SB to the sclera, as strabismus was only noted in this cohort. The numbers in this cohort were significantly smaller and thus the decision to include PPV in the entire cohort was made. The most prominent limitation of the current study is the lack of descriptive and quantitative characteristics of the strabismus and techniques with which it was evaluated and corrected. Perhaps most of the strabismus was a specific alignment and thus more information could have been gleaned and the etiology better understood. The mean follow-up of the eyes was roughly one year, so certainly some of the patients with strabismus may have resolved without intervention if followed long enough, albeit less likely by a year. Additionally, the incidence of preoperative strabismus was not recorded, but only patients with new strabismus following SB surgery were recorded as having postoperative strabismus. Certainly, knowing the preoperative status may strengthen the study, but would not have changed the rate of new postoperative strabismus following the surgery.

In summary, regardless of the method of securing encircling SBs during retinal detachment repair, there were no differences in the rate of anatomic success. The use of either tunnel or suture is entirely surgeon dependent, both of which provide similar visual and anatomical outcomes. The use of scleral tunnels was associated with a higher risk of postoperative strabismus, but not when accounting for multiple variables, in eyes treated with a combination of vitrectomy and scleral buckling. The etiology is unclear, but perhaps scleral tunnels may be associated with a higher rate of postoperative strabismus. Further studies to elucidate this potential association are warranted.

##  Financial Support and Sponsorship

This study was supported by the Phillips Eye Institute Foundation and the VitreoRetinal Surgery Foundation.

##  Conflicts of Interest

Consultant for Alcon (DE, OG, YY), Grant support from Alcon (AC), Royalties from Alcon (EHR), Stockholder in Aldeyra Therapeutics (DE), Consultant for Dutch Ophthalmic (DE), Scientific Advisory Board for Pykus Therapeutics (DE), Stockholder in Valiant and Glaukos (GGE).
